# A PDMS-Based Microfluidic Hanging Drop Chip for Embryoid Body Formation

**DOI:** 10.3390/molecules21070882

**Published:** 2016-07-06

**Authors:** Huei-Wen Wu, Yi-Hsing Hsiao, Chih-Chen Chen, Shaw-Fang Yet, Chia-Hsien Hsu

**Affiliations:** 1 Institutes of Biomedical Engineering and Nanomedicine, National Health Research Institutes, Zhunan 35053, Taiwan; fecancer@gmail.com; 2 Institute of Nano Engineering and MicroSystems, National Tsing Hua University, Hsinchu 30013, Taiwan; top.study@kimo.com (Y.-H.H.); chihchen@mx.nthu.edu.tw (C.-C.C.); 3 Department of Power Mechanical Engineering, National Tsing Hua University, Hsinchu 30013, Taiwan; 4 Institute of Cellular and System Medicine, National Health Research Institutes, Zhunan 35053, Taiwan; syet@nhri.org.tw

**Keywords:** microfluidic hanging drop, embryonic stem cell, embryoid body

## Abstract

The conventional hanging drop technique is the most widely used method for embryoid body (EB) formation. However, this method is labor intensive and limited by the difficulty in exchanging the medium. Here, we report a microfluidic chip-based approach for high-throughput formation of EBs. The device consists of microfluidic channels with 6 × 12 opening wells in PDMS supported by a glass substrate. The PDMS channels were fabricated by replicating polydimethyl-siloxane (PDMS) from SU-8 mold. The droplet formation in the chip was tested with different hydrostatic pressures to obtain optimal operation pressures for the wells with 1000 μm diameter openings. The droplets formed at the opening wells were used to culture mouse embryonic stem cells which could subsequently developed into EBs in the hanging droplets. This device also allows for medium exchange of the hanging droplets making it possible to perform immunochemistry staining and characterize EBs on chip.

## 1. Introduction

Owing to their ability to self-renew and differentiate into any cell type in the body, embryonic stem cells (ESCs) have been widely studied for potential uses in tissue engineering and cell therapy [1,2,3]. When the factors that maintain the stemness of ES cells are removed, ES cells spontaneously aggregate into a 3-dimensional sphere called embryonic body (EB) which includes all three germ layers of endoderm, mesoderm, and ectoderm, and can be induced to differentiate into different cell types [2,3,4,5,6]. Many cell culture approaches have been applied to forming EBs, including suspension culture in bacterial-grade dishes [7,8,9,10], culture in methylcellulose semisolid media [10,11], suspension culture in low-adherence vessels [12,13,14], culture in spinner flask [15] and vessel bioreactor [16]. When cultured in bacterial-grade dishes, the ES cells can adhere to each other spontaneously and not attach to the plastic surface [7,8,9,10]. However, the size and shape of the EBs are heterogeneous, thus they lose synchrony during differentiation. When ES cells are cultured in methycellulose semisolid media [11,17], each single ES cell can be isolated in the methycellulose matrix and develop into a EB. The disadvantage of this technique is that handling semisolid solution by pipettes is difficult. ES cells can also be cultured in low-adherence vessels. Some reagents such as proteoglycan [12], pluronic [13] and 2-methacryloyloxyethyl phosphorylcholine (MPC) [14] were coated on the dish or plate to prevent cell adhesion so that the ES cells can aggregate into EB spheres. Culturing ES cells in spinner flasks or vessel bioreactors [14,16] is preferable for scalable culture of EBs, but the culture conditions (e.g., cell density, hydrodynamic force etc.) of the devices need to be carefully optimized as they can affect the proliferation and differentiation of the EBs. Recently, microfabricated devices containing microchannel [18,19,20] and microwells [21,22,23] have also been used for EB formation. They represent an attractive approach due to their ability to precisely control the size of the formed EBs which can be easily observed on the devices. 

Despite having a wide selection of the aforementioned methods to choose from, currently the conventional hanging drop method is still the most widely used approach for EB formation because it is easy to perform in the laboratory and has minimal equipment and material requirements [24]. Briefly, this method uses a manual pipette to drip individual small droplets of ES cell-containing medium on a Petri dish lid, followed by inverting the lid to hang the droplets, in which the cells spontaneously fall into the bottom of the droplets by gravity and gather at the apexes due to the concave shape of the droplets. The size of EB can be controlled by adjusting the density of the cell suspension being used for forming the droplet. The uniqueness of this method lies in the fact that the cells are cultured at the liquid-air interface of the droplet thus eliminating cell contact with a solid substrate which otherwise needs a treatment to become non-adherent to cells in other methods. However, the hanging droplet method is limited by the difficulty of exchanging medium in the droplets and the required manual operation for each individual droplets. Previously, a hollow sphere soft lithography approach was used to make a novel device capable of hosting 500 μL medium in each hanging droplet, thus allowed the medium to be exchanged for EB culture for more than 10 days [25]. However, the throughput of the process is still limited because each droplet has to be individually handled. To solve this problem, a modified 384-well plate device has been developed for hanging drop culture which can be scaled up by the assistance of a robotic arm system [26]. 

In this paper, we report the development of a microfluidic chip-based method for hanging droplet EB culture. Our approach utilizes a microstructure design (microchannel with opening wells) and microfluidic controls to achieve high-through culture of the cells. In our proof-of-concept experiment, we designed and fabricated a 6 × 12 arrayed micro-hanging drop chip in which a large number of hanging droplets can be simultaneously formed without needing to pipette the droplets individually. We have also developed a setup and operation procedure for successful on-chip culture of EBs as demonstrated by their growth curve and expressions of pluripotency markers.

## 2. Results

### 2.1. Chip Design and Experimental Process and Setup

**Figure 1:**
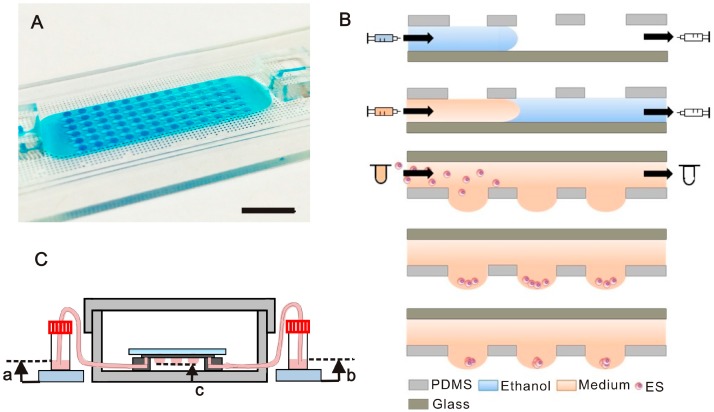
Illustration of the microfluidic hanging drop chip, and its setup and operation. (**A**) Photo of the PDMS μHD chip (openings facing up) with opening diameter of 1000 μm. Scale bar is 1 cm; (**B**) Schematic illustration of the μHD chip operation. The microchannel is firstly filled with 75% ethanol, followed by introducing cell suspension into the microchannel by hydrostatic pressure-driven flow, and allowing cells to descend to the bottom of the droplets. The docked cells centralized and aggregated at center of droplet bottom due to the concavity of the droplets and grow into spheroids; (**C**) Illustration of μHD chip setup. The μHD chip was placed in a 10 cm dish containing 1× PBS in the bottom of the plate to prevent medium evaporation from the hanging droplets.

The arrayed microfluidic hanging drop (μHD) device is composed of PDMS microfluidic channels with 6 × 12 opening wells on a glass slide as shown in Figure 1A. The microchannels are 1200 μm wide and 120 μm tall. The opening wells are 1000 μm in diameter and 130 μm tall (please see Supplementary Figure S1 for the detailed design parameters). The entire operation was performed inside a laminar flow hood to keep the device sterile. The chip’s inlet hole and outlet hole was initially connected to a syringe with 75% ethanol solution and an empty syringe respectively. As shown in Figure 1B, the ethanol solution was then introduced into the microchannel by manually pushing the inlet syringe while withdrawing the outlet syringe. The inlet syringe was then replaced with a cell culture medium-containing syringe to replace the ethanol solution suing the same manual push and withdraw procedure. Subsequently, the inlet tubing was unplugged and replaced with a tubing connected to an Eppendorf tube containing embryonic stem cells suspension at 3 × 10^5^ cell/mL concentration, whereas the outlet tubing was unplugged and replaced with another tubing connected to a plastic tube containing 100 μL medium. The chip was then flipped to have the openings facing down, placed in the Petri dish, and moved to the stage of a microscope to observe the microchannel area when cells were being introduced into the chip. The ES cell suspension was injected into the microchannel by using hydrostatic pressure, which was generated by elevating the cell suspension tube for 30 cm and lowering the outlet tube for 30 cm with respect to the level of the chip. Note that the flipped chip was supported by two PDMS blocks in a way that there is a ~0.4 cm distance between the chip and the dish to avoid potential contact between the substrate and the formed droplets. The dish was also added with 4 mL of 1× PBS solution and covered with a lid to prevent medium evaporation. The duration of the cell suspension loading step was 15 min, which allowed the cell suspension to fill the microchannel and the cells to fall into the wells. Subsequently, the cell suspension tube was replaced with a cell medium-containing reservoir, followed by adjusting both the inlet and outlet medium reservoirs to the same height (Figure 1C, a = b = 1.45 cm) to stop the flow and apply a hydrostatic pressure needed for maintaining the concave droplet shape for EB formation (Figure 1C). The device was then placed into a cell culture incubator to allow the ES cells to aggregate in the droplets. Note that after 3 h, the height of the medium reservoirs was lowered to 1.4 cm (a = b = 1.4 cm) to reduce the hydrostatic pressure, thus the chance of bursting the droplets during the following cell culture time. The cell culture medium was replaced daily. To replace medium in the microchannel, 100 μL of medium in the inlet tube was taken out followed by adding 100 μL of fresh medium into the tube. Then 100 μL of medium in the outlet tube was taken out and the outlet tube was lowered to 0.2 cm medium high (b = 0.2 cm) to generate a hydrostatic pressure-driven flow from the outlet to inlet tube, thus medium exchange in the microchannel. This setup is kept for 2 h (inside a cell culture incubator), before a total of 100 μL medium was added into both tubes adjust the medium level s back to the same height of 1.4 cm (a = b = 1.4 cm).

### 2.2. Characteristics of the Microfluidic Device

#### 2.2.1. Hanging Drop Formation

**Figure 2:**
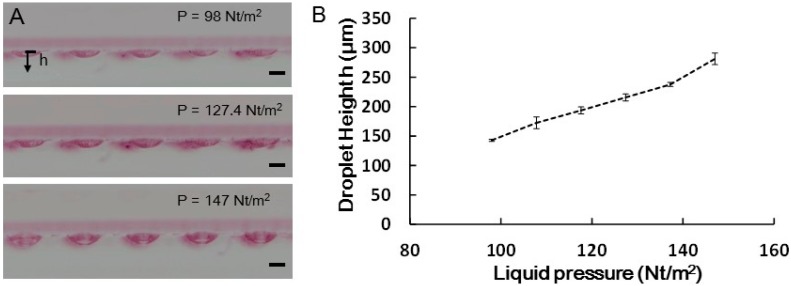
Measured droplet heights under various hydrostatic pressures in μHD chip with 1000 μm diameter opening wells. (**A**) Side-view photographs of a portion of the μHD chip showing protruding droplets of different heights (top: average height = 142.8 μm at 98 N/m^2^; middle: average height = 215.6 μm at 127.4 N/m^2^; bottom: average height = 281.2 μm at 147 N/m^2^; (**B**) the relationship between the droplet height and hydrostatic pressure. Scale bar is 500 μm.

In order to control droplet formation, droplet height under various hydrostatic pressures were measured in μHD chip with 1000 μm diameter opening wells. Figure 2A show the side view of the formed water droplets at the bottom of the microchannel. The relationship between the droplet height and applied hydrostatic pressure is shown in Figure 2B. In our tested pressure range, we found that the formed droplet height is linearly proportional the hydrostatic pressure and the maximum droplet height of ~281 μm is achieved under a hydrostatic pressure of 147 N/m^2^, above which the formed droplets are less stable and prone to bursting. For our cell culture experiment, a hydrostatic pressure of 98 N/m^2^ was chosen, because it was large enough to form a proper concave droplet shape for cells to concentrate at the droplets’ apexes and at the same time allow the droplets to be more robust. 

#### 2.2.2. Demonstration of Solution Exchange in μHD Chip

**Figure 3:**
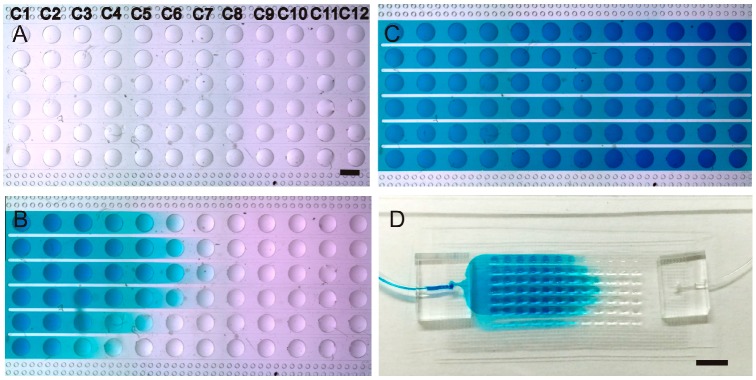

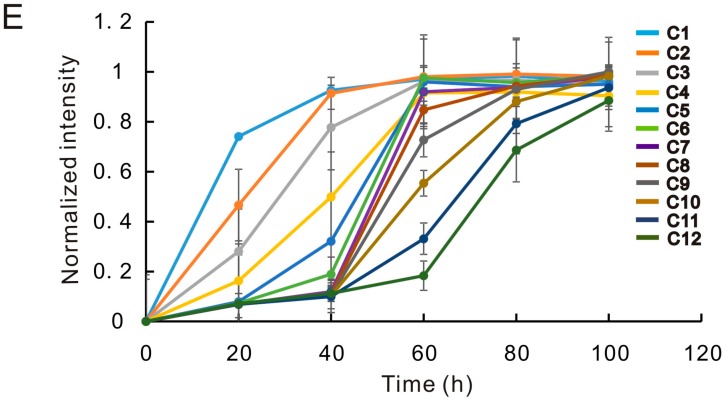
Demonstration and characterization of solution exchange in μHD chip. (**A**–**C**) Photographs showing the solution replacement process (blue dye replacing DI water) at 0 min, 60 min and 100 min, respectively. Scale bar = 1 mm; (**D**) photograph of the whole chip taken at 80 min after the replacement process started. Scale bar = 4 cm; (**E**) the relationship between the normalized intensity of the blue dye solution and the time after the replacement process started.

One of the limitations of the hanging drop method is the difficulty in exchanging medium of the droplets. The use of microchannels makes it easy to exchange medium in the hanging droplets in the μHD chip. To demonstrate and characterize this process, the μHD chip was firstly set up with the hydrostatic pressure driving flow setup with both reservoirs filled with water. The height difference between the inlet reservoir and outlet reservoir was set to be 1.2 cm to produce a hydrostatic pressure-driven flow from inlet to outlet, followed by adding 20 μL of blue dye solution into the inlet reservoir in order to observe the advancement of the flow to measure the time needed for replacing the solution in the μHD chip. The top-view images (Figure 3A–C) shows the progress of the front line of the blue dye solution in the microchannel at 0 min, 60 min, and 100 min time points after the blue dye was added to the inlet reservoir. The result shows that the water in the microchannel can be replaced by the blue dye solution in 100 min. 

### 2.3. EB Formation

**Figure 4:**
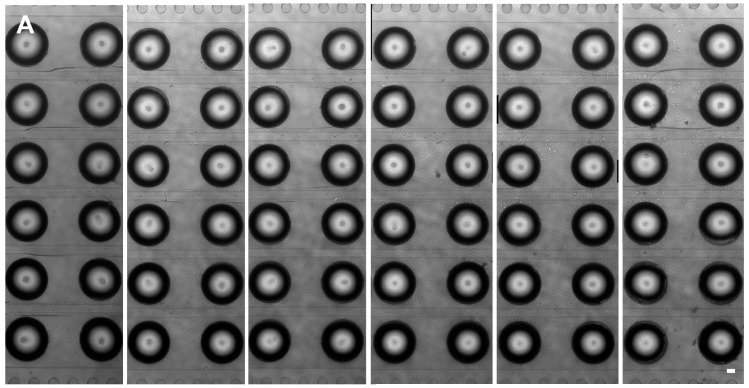

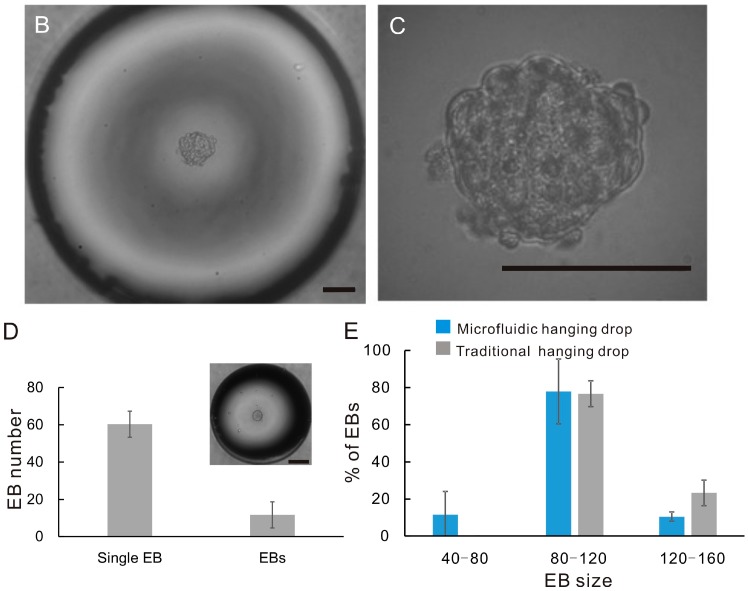
EB formation after 1 day. (**A**) Images of the top-view of the chip after 1 day culture; (**B**,**C**) The closed view of one EB; (**D**) the comparison between the number of single EB and EBs. The photo shows the satellite EBs are less than 40 μm; (**E**) the size distribution of the EBs.

For EB formation, ES cells were introduced into the microchannel, followed by elevating the inlet and outlet medium reservoirs to produce a hydrostatic pressure for maintaining the concave droplet shape for the ES cells to aggregate. In our study, cell suspension at 3 × 10^5^ cells/mL density was introduced into the microchannels, resulting in 150~400 cells in each well. Figure 4A shows the images of EBs formed in 72 hanging droplets at the 72 opening wells after 1 day of culture. Due to the concave shape of the droplets, the formed EBs were located at the center of each well (Figure 4A,B). It was observed that some droplets could contain more than one EB in it. Therefore, we have also calculated the number of EB in the droplets. As shown in Figure 4D, 83.8% of the wells had a single EB in each well, whereas the rest (16.2%) of the wells contained a single EB with satellite EBs whose size were less than 40 μm (>98%). The size of the single EBs was calculated by measuring their diameters using the ImageJ software. The mean diameter (R) of the EB was determined according to the following equation: R = (a × b)^1/2^, where a and b are two orthogonal diameters of the EB. Figure 4E shows the size distribution of the formed EBs in the opening wells. The percentage of EBs was 11.6%, 77.9%, 10.5% in the diameter range of 40~80 μm, 80~120 μm, 120~160 μm, respectively. The data shows that most of the EBs were 80 to 120 μm in diameter. The EBs size was comparable to that of EBs formed by using conventional hanging drop method (Figure 4E). The average coefficient of variance (CV) for the diameter of the EBs is calculated about 15.9%. EBs were then observed after 1 day to 3 day.

**Figure 5:**
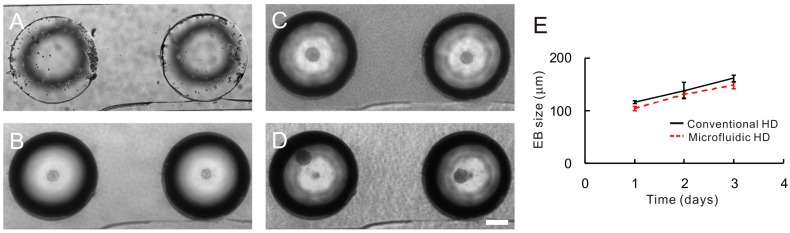
EB growth observation for 3 days. (**A**–**D**) Images of EBs taken at day 0 (**A**); day 1 (**B**); day 2 (**C**) and day 3 (**D**) after cell seeding; (**E**) the growth curves of the EBs cultured in the μHD chip and conventional hanging drop system. The scale bar is 200 μm.

Figure 5A–D shows the images of the EBs formation in four wells during three days of culture. The ES cells formed EBs which continued to be proliferated. The sizes of EBs were measured every day to obtain the growth rates of the EBs. Figure 5E shows the growth curves of EBs cultured in μHD chip and conventional system. The EBs in the μHD system had a growth similar to that of EBs cultured by the conventional method.

### 2.4. Immunostaining of EBs

**Figure 6:**
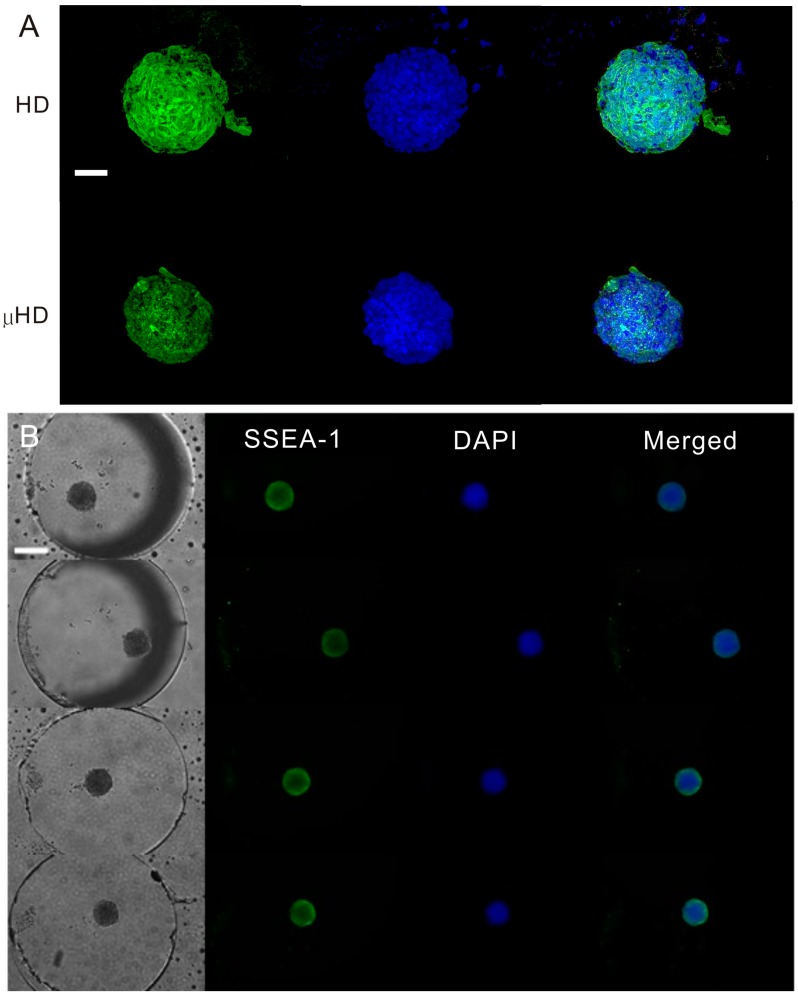
Immunochemistry staining of EBs. (**A**) Confocal images of SSEA-1 and DAPI stained EBs formed by using the conventional hanging drop method and the μHD chip. Scale bar = 50 μm; (**B**) images of on-chip immunochemistry stained EBs. Scale bar = 100 μm.

After three days of EB culture, 500 μL of medium was manually injected into the microchannel to burst the droplets and collect the EBs in a Petri dish. The EBs were then stained with SSEA-1 antibody and 4′,6-diamidino-2-phenylindole (DAPI). Figure 6A shows confocal images of immunostained EBs formed by conventional hanging drop method and the microfluidic chip. The positive SSEA-1 green fluorescence staining indicates that the pluripotency of the EBs were maintained in EBs cultured by both methods. The DAPI staining was used to visualize the nucleus of the cells. Note that because the μHD chip allows solution exchange in hanging droplets, we were also able to perform the immunochemistry staining of EBs on chip (Figure 6B). This unique feature of the μHD chip can eliminate cell transferring steps required in the conventional hanging drop method, thus potential cell loss and damage. 

## 3. Materials and Methods

### 3.1. Fabrication Process

The microfluidic device was fabricated in polydimethylsiloxane (PDMS) formed from mixing prepolymer (Sylgard 184, Dow Corning, Auburn, MI, USA) at a ratio of 1:10 base to curing agent using a modified soft lithography method. Briefly, a master mold with two-layer features were made using conventional photolithography with SU-8 (MicroChem, Newton, MA, USA) on a silicon wafer. The channel (layer 1) were 1200 μm in width, 120 μm in height, and the extruding feature was 130 μm tall cylinders (layer 2) with 1000 μm diameter. The master was then used as mold and placed on a thin layer of PDMS sheet on a glass plate. PDMS prepolymer was then poured on to the master followed by applying a fluoropolymer coated polyester (PE) sheet (Scotchpak 1022, 3M, St. Paul, MN, USA) and a glass plate to sandwiched the master in between the two glass plates which served to apply even pressure to squeeze out excess PDMS. The thin PDMS was then cured at 65 °C for 24 h and then removed from the master with the PE sheet without distortion[27]. Then the membrane was bond onto a glass slide after it was treated with oxygen plasma (AP300, Nordson March, Westlake, OH, USA). The PE sheet was then removed from the PDMS. Two PDMS blocks (10 mm × 10 mm × 2 mm) with punched holes (I.D. = 0.75 mm) were bonded to the PDMS to serve as inlet and outlet for connecting to tubing (I.D. = 0.01 inch, AAQ04091 Tygon Microbore Tubing, Buckeye Container Co., Wooster, OH, USA). Prior to use, the devices were sterilized by the UV light for 30 min. 

### 3.2. Droplet and EB Measurement

As shown in Supplementary Figure S2, the chip was connected to two plastic reservoirs (id 14.5 mm, 6 cm high, 5 mL) via two tubings (I.D. = 0.01 inch, AAQ04091 Tygon Microbore Tubing) and placed on two PMMA plates under a microscope (Nikon SMZ1500, Buckeye Container Co., Wooster, OH, USA) equipped with a CCD camera (Qimaging, Surrey, BC, Canada). A mirror (5 cm × 7 cm) was set beside the chip and tilted with a 45° angle so that the side view of the droplets could captured by the CCD camera through the microscope. Droplet images were captured from three experiments and from each image the heights of 5 droplets were measured using a software (ImageJ, NIH, Bethesda, MD, USA). The exerted hydrostatic pressure at the opening is calculated by using the hydrostatic pressure equation as: p=ρ g H, where p is pressure (N/m^2^), ρ is density of liquid (water: 1000 kg/m^3^), g is acceleration of gravity (9.8 m/s^2^), and H (H = A − C) is height of fluid (m). The images of cells in the μHD chip was captured by using an inverted fluorescence microscope (Eclipse Ti, Nikon, Melville, NY, USA) equipped with a CCD camera (RT3, Spot, Sterling Heights, MI, USA).

### 3.3. Medium Exchange Experiment

The degree of solution replacement in the microchannel was evaluated by the intensity of the blue dye measured in the droplets areas. Specifically, the images were firstly converted to 8-bit color images and then to gray scale images. The function of Image Calculator in ImageJ was then used to substrate every image with the background image (i.e., the image taken at 0 min). The gray value at the well areas were then measured from the calculated images. The gray scale value of 0 was regarded as 0% of blue dye replacement whereas the maximum number was regarded as 100% blue dye replacement. The normalized intensity was obtained by dividing the gray value of interest to the maximum gray scale value. The wells were divided into 12 columns (six wells are in one column, C1 to C12 from the inlet side to the outlet side of the chip), and the averaged normalized intensities of each column was plotted. Three experiments were repeated. 

### 3.4. mES Cell Culture, Cell Line: D3

In this study, mouse embryonic stem cells (mESCs; ES-D3) cultured on mouse embryonic fibroblast (MEF) (M-EF001, BCRC, Hsinchu, Taiwan) feeder were used to prepare ES cell suspension. The ES-D3 on the feeder was cultured in medium consisting of DMEN with 15% *v*/*v* FBS (SH30070, Thermo HyClone, Waltham, MA, USA), 1% *v*/*v* non-essential amino acids (NEAA) (Gibco Invitrogen Co., Waltham, MA, USA), 1% *v*/*v* l-glutamine (Gibco Invitrogen Co.), 0.1 mM 2-mercapto-ethanol (Gibco Invitrogen Co.) and 1000 U/mL ESGRO (ESG1107, Millipore, Billerica, MA, USA) which contains leukemia inhibitory factor (LIF). For preparing the feeder, the MEFs were cultured in Dulbecco’s modified eagle’s medium (DMEM) (Gibco Invitrogen Co.) with 10% *v*/*v* FBS. Cultured ES cells were treated with collagenase IV (Gibco Invitrogen Co.) to recover them from the MEF feeder, followed by dissociation with TrypLE (Gibco Invitrogen Co.) to obtain ES cell suspension prior to hanging droplet culture experiment. 

### 3.5. Immunochemistry Staining

The on-chip immunochemistry staining was performed in the steps illustrated in Supplementary Figure S3. To load the solutions into the microchannel, the outlet reservoir tube was adjusted to be 3.4 cm lower than the inlet reservoir tube to generate a hydrostatic pressure difference to drive the solution. First, phosphate buffered saline (PBS, Invitrogen, Waltham, MA, USA) was loaded in the inlet reservoir to replace the medium in the microchannel for 10 min, followed by replacing the PBS reservoir with a reservoir containing 4% formalin to fix the EBs for 30 min at room temperature. To permeabilize the cells, 0.5% Triton X-100 (Sigma-Aldrich, St. Louis, MO, USA) was loaded into microchannel for 25 min. After a 10 min wash with 1X PBS for 10 min, primary antibody Stage-specific embryonic antigen-1 (SSEA-1, Millipore) diluted in 3% bovine serum albumin (BSA) and 0.1% Triton X-100 (1:200) was loaded into the chip and incubated for 1 h in an incubator at 37 °C. After washing with PBS for 1.5 h, the cells were incubated with a secondary antibody of 1:200 dilution of anti-mouse IgG conjugated with fluorescin isothiocyanate (FITC, Millipore). 4′,6-Diamidino-2-phenylindole (DAPI, Sigma-Aldrich) at 1:1000 in PBS was then loaded to fluorescently label the cell nuclei for 30 min at room temperature. The EBs were then washed with PBS and observed under a fluorescent microscope. For immunochemistry staining of EBs formed by traditional hanging drop method, the harvested cells were washed three times in PBS, fixed with 4% paraformaldehyde at room temperature for 30 min, permeabilized with 0.5% Triton-X 100 in PBS at room temperature for 15 min, incubated with 3% BSA in 0.1% Triton-X 100 at 4 °C for 1 h and then washed three times with PBS. The cells were subsequently incubated with primary antibodies in 0.1% Triton-X 100 for overnight at 4 °C, washed three times with PBS, incubated with the fluorescently labeled secondary antibody for 2 h at room temperature and finally washed three additional times with 1XPBS.

## 4. Conclusions

We have developed a microfluidic chip-based system for EB formation in hanging droplets. Our device represents an alternative method for high-throughput EB formation without using complex equipment. This method also allows for medium exchange during EB culture via the microfluidic channel so it may be used for direct characterization of the cultured EBs on chip. However, some improvements are still required to make this method more practical in the future. First, the higher (as compared to the conventional hanging drop method) size variance of the microfluidic chip-formed EBs needs to be reduced. This is due to the fact that our current device fabrication method involves several manual procedures (e.g., inlet hole pouncing, and clamp modeling), which resulted into device variation in terms of inlet hole position and PDMS channel ceiling thickness. From our observation, in the cell seeding step the offset of the inlet hole could affect the lateral distribution of the incoming cells whereas PDMS ceiling thickness variation could cause non-uniform PDMS channel bulging which resulted into various flow conditions even when the same hydrostatic pressure was used. We envision that the cell seeding number uniformity could be improved by improving the fabrication method and the material of the microfluidic device. Second, our current device operation method involves several manual steps to switch solutions which are tedious and could be difficult to operate for a regular laboratory personnel. It would be necessary to reduce the amount of manual operation (e.g., by integrating fluid control components into the chip) in order to disseminate this new methods to more applications where cell aggregates culture is needed.

## Supplementary Materials

Supplementary materials can be accessed at: http://www.mdpi.com/1420-3049/21/7/882/s1.

## Abbreviations

The following abbreviations are used in this manuscript:

ESCs	Embryonic stem cells
EB	Embryoid body
BSA	Bovine serum albumin
CV	Coefficient of variance
DAPI	4′,6-Diamidino-2-phenylindole
DMEM	Dulbecco’s modified eagle’s medium
EC	Embryonic carcinoma
FITC	Fluorescin isothiocyanate
MEF	Mouse embryonic fibroblast
MPC	2-Methacryloyloxyethyl phosphorylcholine
mESCs	Mouse embryonic stem cells
NEAA	Non-essential amino acids
PBS	Phosphate buffered saline
PE	Polyester
PDMS	Polydimethylsiloxane
SSEA-1	Stage-specific embryonic antigen-1
LIF	Leukemia inhibitory factor
μHD	Microfluidic hanging drop
