# Supplementary material for: A PDMS-Based Microfluidic Hanging Drop Chip for Embryoid Body Formation
Source: Molecules. 2016 Jul 6;21(7):882. doi: 10.3390/molecules21070882 (PMC6272923; doi:10.3390/molecules21070882)
Supplement: Supplementary file 1 [file molecules-21-00882-s001.pdf]

Supplementary Materials: A PDMS-Based Microfluidic Hanging Drop Chip for Embryoid Body Formation

Huei-Wen Wu, Yi-Hsing Hsiao, Chih-Chen Chen, Shaw-Fang Yet and Chia-Hsien Hsu

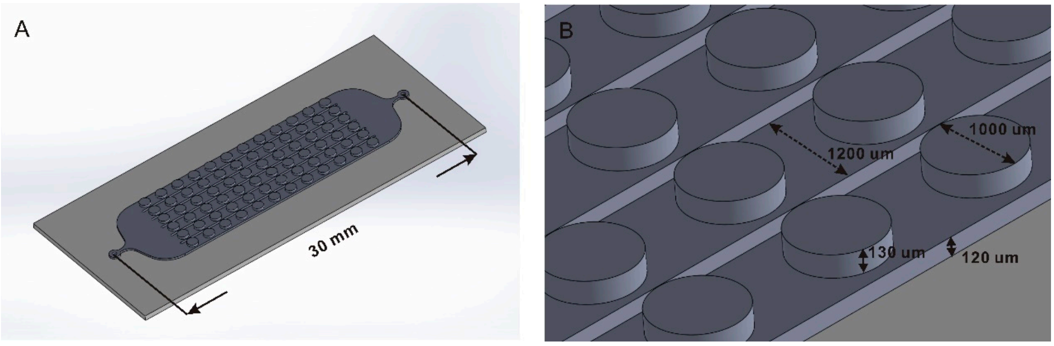

Figure S1. 3D illustration of the SU-8 mold. (**A**) Full mold; (**B**) Close-view of the mold.

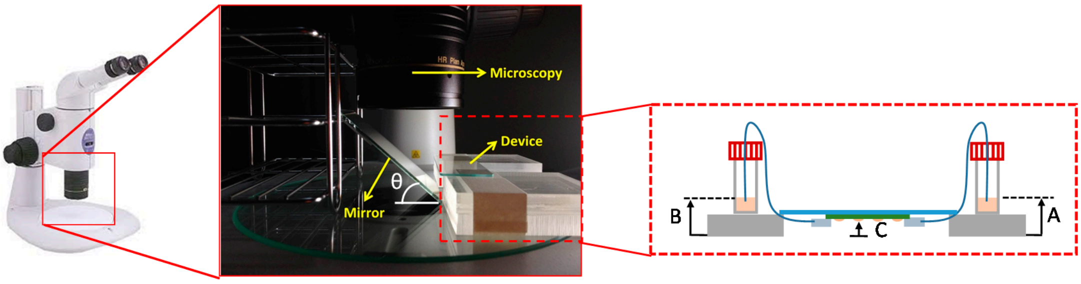

Figure S2. Setup of the droplet measurement. **A** and **B** are the combined height of medium and PMMA plate from the microscope stage, whereas C is the distance from the droplet's bottom to the microscope stage.

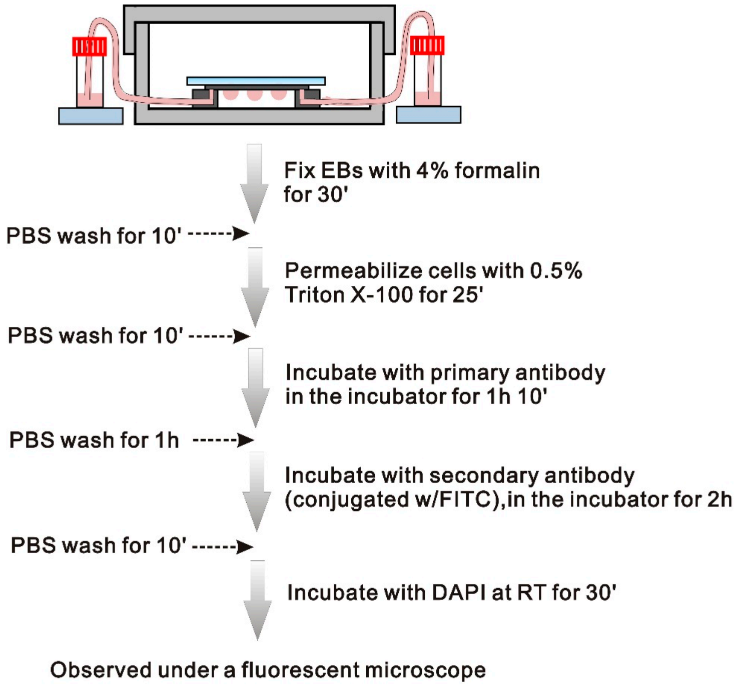

Figure S3. Illustration of on-chip immunochemistry staining steps.
